# Improved reconstruction and comparative analysis of chromosome 12 to rectify Mis-assemblies in *Gossypium arboreum*

**DOI:** 10.1186/s12864-020-06814-5

**Published:** 2020-07-08

**Authors:** Javaria Ashraf, Dongyun Zuo, Hailiang Cheng, Waqas Malik, Qiaolian Wang, Youping Zhang, Muhammad Ali Abid, Qiuhong Yang, Xiaoxu Feng, John Z. Yu, Guoli Song

**Affiliations:** 1 Institute of Cotton Research, Chinese Academy of Agricultural Sciences, Anyang, 455000 China; 2 Genomics Lab, Department of Plant Breeding and Genetics, Faculty of Agricultural Sciences and Technology, Bahauddin Zakariya University, Multan, Punjab 60000 Pakistan; 3 Zhengzhou Research Base, State Key Laboratory of Cotton Biology, Zhengzhou University, Zhengzhou, 450001 China; 4 College of Life Sciences, Tarim University, Alar, 843300 China; 5 Crop Germplasm Research Unit, Southern Plains Agricultural Research Center, US Department of Agriculture–Agricultural Research Service (USDA-ARS), College Station, TX 77845 USA

**Keywords:** Genetic map, Reference-assisted assembly, Syntenic relationship, Gene loss, Transcription factor

## Abstract

**Background:**

Genome sequencing technologies have been improved at an exponential pace but precise chromosome-scale genome assembly still remains a great challenge. The draft genome of cultivated *G. arboreum* was sequenced and assembled with shotgun sequencing approach, however, it contains several misassemblies. To address this issue, we generated an improved reassembly of *G. arboreum* chromosome 12 using genetic mapping and reference-assisted approaches and evaluated this reconstruction by comparing with homologous chromosomes of *G. raimondii* and *G. hirsutum*.

**Results:**

In this study, we generated a high quality assembly of the 94.64 Mb length of *G. arboreum* chromosome 12 (A_A12) which comprised of 144 scaffolds and contained 3361 protein coding genes. Evaluation of results using syntenic and collinear analysis of reconstructed *G. arboreum* chromosome A_A12 with its homologous chromosomes of *G. raimondii* (D_D08) and *G. hirsutum* (AD_A12 and AD_D12) confirmed the significant improved quality of current reassembly as compared to previous one. We found major misassemblies in previously assembled chromosome 12 (A_Ca9) of *G. arboreum* particularly in anchoring and orienting of scaffolds into a pseudo-chromosome. Further, homologous chromosomes 12 of *G. raimondii* (D_D08) and *G. arboreum* (A_A12) contained almost equal number of transcription factor (TF) related genes, and showed good collinear relationship with each other. As well, a higher rate of gene loss was found in corresponding homologous chromosomes of tetraploid (AD_A12 and AD_D12) than diploid (A_A12 and D_D08) cotton, signifying that gene loss is likely a continuing process in chromosomal evolution of tetraploid cotton.

**Conclusion:**

This study offers a more accurate strategy to correct misassemblies in sequenced draft genomes of cotton which will provide further insights towards its genome organization.

## Background

A high-quality genome sequence of species is a prerequisite to provide an inclusive access to complete genes catalog, different regulatory elements controlling their functions, and provides a framework for exploring genomic variations. During the early stages of genome sequencing, capillary technique was used to sequence the free-living organisms, starting with simple microbial genomes [1] followed by plant genomes including *Arabidopsis thaliana* [2], *Oryza sativa* [3] and *Carica papaya* [4]. Afterwards, many other complex plant genomes have been sequenced [5–8] using next-generation sequencing techniques (NGS). In current era, long-read sequencing (LRS) holds the promises due to its long-reads lengths [9], and many complex plants genome have been sequenced by this technique [10, 11].

In contrast to significant improvement of sequencing techniques, genome assembling continues to encounter many challenges [12, 13]. Particularly, complex and large plant genomes have remained a great challenge for de novo assembly due to its large genome size [14], high ploidy level [15], high rate of repeat elements [16], complex gene contents and high transposon’s activities [17]. One of the most difficult problems during *de-novo* genome assembly is the ordering and orientation of scaffolds to reconstruct the pseudo-chromosomes. A vigorous de novo assembly of chromosomes requires good quality physical and genetic maps [18, 19], optical maps [20], Hi-C sequence data [21] and genome collinearity and synteny [22] to anchor and orient the scaffolds to reconstruct the chromosomes. However, lack of good genetic or physical maps for most of the newly sequenced species makes difficult the accurate ordering of scaffolds into chromosomes. In this situation, good quality sequenced and assembled “reference genome” of closely related species would guide to an alternative approach which is referred as reference-assisted chromosome assembly. Orientation of scaffolds into chromosomes by reference-assisted chromosome assembly helps to exploit the benefits of assembled chromosomes without adding further efforts of sequencing or map construction [23].

Cotton (*Gossypium* spp.) is an important natural fiber and edible oil crop, mainly grown in subtropical and temperate areas of the world. Tetraploid genome of cotton is complicated by the presence of two sub-genomes (A_T_ and D_T_) in its nucleus which were derived from diploid A-genome (*G. arboreum*) and D-genome (*G. raimondii*) progenitors. Diploid A genome is about 2-fold larger than D progenitor genome, and A_T_ sub-genome is more stable in *G. hirsutum* than D_T_ sub-genome [24]. Furthermore, *G. arboreum* possesses valuable and unique traits such as early maturity, tolerance to biotic and abiotic stresses and great fiber strength, providing a valuable germplasm resource for improving modern tetraploid cotton cultivars [25]. Therefore, existence of high quality reference draft genome sequence of *G. arboreum* is an essential task for tracing the origin of genome segments and interference of homoeology i.e. genes and RNA-seq [26] in tetraploid cotton.

Previously, genome of cultivated diploid cotton *G. arboreum* (Shixiya1) was sequenced and assembled using whole-genome shotgun approach which contained a total of 1694 Mb length including 41,330 protein coding genes and 1145 Mb long terminal repeats (LTR)-type retrotransposons [27]. Subsequently, genome sequence of tetraploid cotton *G. hirsutum* [28] was released which showed a conserved gene order with the A cotton genome (*G. arboreum*) [27]. However, another sequenced version of *G. hirsutum* genome [8] reported unobvious collinearity with the sequenced genome of *G. arboreum* [27], which is mainly due to numerous misassemblies in *G. arboreum* genome [27]. For instance, several scaffolds belong to different chromosomes were present in one pseudo-molecule of *G. arboreum*. Several previous studies reported that draft sequenced genome of *G. arboreum* [27] contained errors and mis-assemblies [8, 29, 30], however this draft genome did not undergo precise quality improvement to correct errors. So, knowing how to assemble this genome accurately, how to best make use of the highly fragmented assemblies and how to perform these applications at the lowest cost are important in today’s funding environment [31]. Here, we demonstrated an initial more accurate effort to reassemble chromosome 12 (A_A12) of *G. arboreum* using NGS data from previous study [27] without adding any other sequencing efforts*,* as its homolgous chromosomes of allotetraploid cotton contain important genes related to male sterility, fiber quality and gland development [32–34]. The advantage of selecting chromosome 12 also includes that it do not show any translocation [8, 35] in diploid and tetraploid cotton species. Subsequently, reassembled *G. arboreum* chromosome A_A12 was compared using collinear and syntenic analysis, whole chromosome alignment and dotplotting with its homologous chromosomes 12 of *G. raimondii* (D_D08) and *G. hirsutum* (AD_A12 and AD_D12) as well as previously assembled *G. arboreum* chromosome 12 (A_Ca9) [27] to support the more accuracy of reconstructed chromosome. Furthermore, we performed different comparative analysis such as gene loss, identification and mapping of transcription factor-related genes within homologous chromosomes 12 (A_A12, D_D08, AD_A12 and AD_D12) of three cotton species including *G. arboreum*, *G. raimondii* and *G. hirsutum*.

## Results

### Re-assembling of *G. arboreum* chromosome 12 (A_A12)

**Fig. 1:**
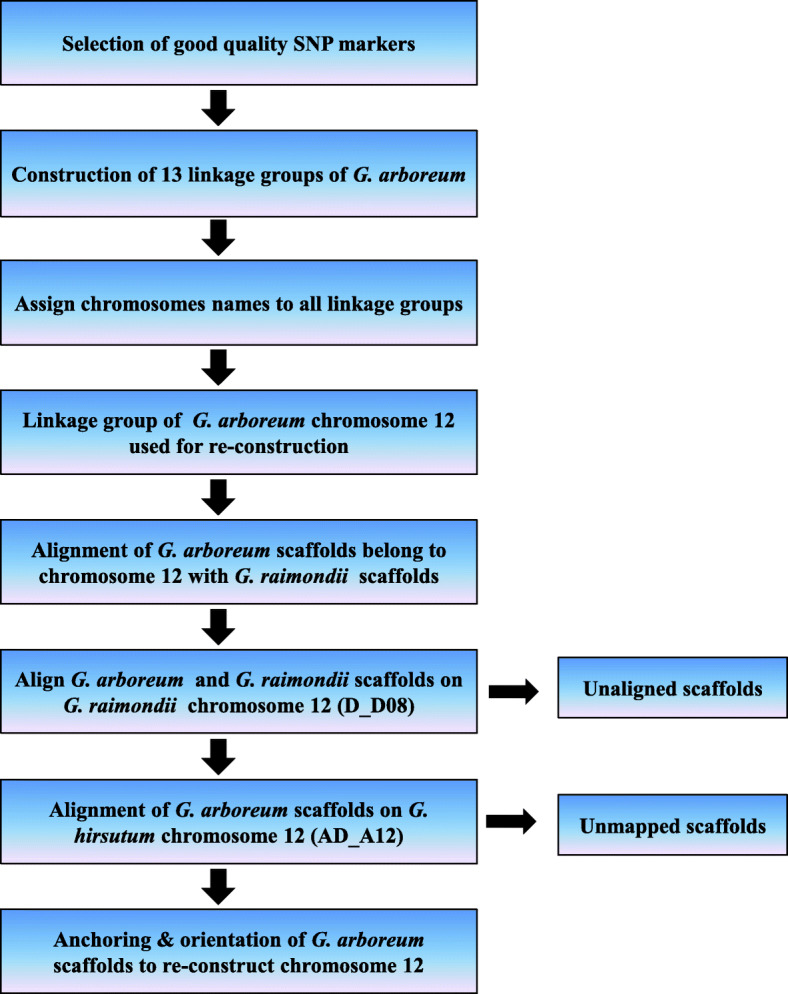
Schematic diagram for reassembling of *G. arboreum* chromosome 12 (A_A12). Each rectangle corresponded to procedures applied for chromosome reassembling steps. Genotypic data of 24,569 SNP markers used in previous study [27] was first filtered out for construction of linkage groups, which were then assigned to 13 chromosomes of *G. arboreum*. Afterwards, linkage group belong to *G. arboreum* chromosome 12 was used for re-assembling. We checked the alignments of scaffolds belonging to *G. arboreum* chromosome 12 for following levels: (i) Alignment of *G. arboreum* scaffolds (obtained by the genetic map) to *G. raimondii* scaffolds [7], (ii) Orientation of *G. raimondii* (obtained from the previous step) and *G. arboreum* scaffolds along *G. raimondii* chromosome (D_D08) [36], and (iii) adjacency of *G. arboreum* scaffolds within *G. hirsutum* chromosome (AD_A12) [8]

Here, we combined genetic mapping and reference-assisted approaches (Fig. 1) to reassemble *G. arboreum* chromosome A_A12.

### Genetic map construction for re-assembling

Initially, 3735 high quality markers were selected out of 24,569 SNPs used in previous study [27] for construction of linkage map. A total of 3544 loci were classified into 13 linkage groups at LOD 06 with a total length of 1599.8 cM. Linkage groups 01 and 02 contained more number of markers as compared to others, while linkage group 13 enclosed lowest number of markers (Additional File 1: Fig. S1, Additional File 2: Table S1). Afterwards, chromosomes names were assigned to 13 linkage groups of *G. arboreum* according to the available mapped markers data of *G. hirsutum* and *G. raimondii* which gave the similar good results (Additional File 2: Table S2 and Table S3). However, we did not get same results in case of using mapped marker data of *G. arboreum* (Additional File 2: Table S4), provided first evidence of misassembles in sequenced genome of *G. arboreum* [27]. After assigning chromosomes names to 13 linkage groups, linkage group belong to *G. arboreum* chromosome 12 (A_A12) was used for further reassembling because it contains important genes for different traits and had no translocation. Final linkage group of *G. arboreum* chromosome A_A12 comprised of 189 markers, distributed within 64 scaffolds and spanned 146.63 cM genetic distance (Additional File 1: Fig. S1, Additional File 2: Table S1).

### Reference assisted approach for reassembling

After construction of genetic map which served as a backbone for subsequent reassembling steps, we assessed *G. arboreum* chromosome A_A12 against two criteria: adjacency of scaffolds and gene integrity via BLAT and gene wise BLASTN approaches (Fig. 1). We checked scaffolds and gene integrity according to three steps: (i) Alignment of *G. arboreum* scaffolds (obtained by genetic map) to *G. raimondii* scaffolds [7], (ii) Orientation of *G. raimondii* (obtained from previous step) and *G. arboreum* scaffolds along *G. raimondii* chromosome D_D08 [36], and (iii) adjacency of *G. arboreum* scaffolds within *G. hirsutum* chromosome AD_A12 [8].

Based on linkage map and reference assisted approaches, we also identified inter-chromosomal mis-assemblies in 08 scaffolds of *G. arboreum* having a total of 19.79 Mb length (Additional File 2: Table S5). The final assembly of *G. arboreum* chromosome A_A12 comprised of 144 scaffolds (N50 = 912 kb) with 94.64 Mb length (Table 1, Additional File 1: Fig. S2).

**Table 1:** Global statistics of reassembled *G. arboreum* chromosome (A_A12)

Category	Statistics
Total length of the assembly (Mb)	94.64
Number of oriented scaffolds	144
Oriented scaffolds (N50) (Mb)	0.912
Maximum scaffold length (Mb)	2.360
Minimum scaffold length (Mb)	0.002
Number of protein coding genes	3361
Average gene size (bp)	2527
Average transcript length (bp)	1263
Gene density (per Mb of chromosome)	36
Total gene region	8,493,379
Total coding Region	3,796,446
Maximum CDS length (bp)	14,331
Average CDS length (bp)	1130
Mean exon number	4.7

### Gene contents of *G. arboreum* chromosome A_A12

We generated an updated list of protein coding genes of reconstructed *G. arboreum* chromosome A_A12 which showed a total of 3361 predicted protein coding genes with an average transcript size of 1263 bp and a mean of 4.7 exons per gene (Table 1). The *Cotton_A_14584* gene contained the largest CDS (14,331 bp) with 13 exons, while smallest CDS (90 bp) was enclosed by *Cotton_A_37648* with 02 exons. Out of 3361 predicted genes, 2456 have predicted functional description. Gene density is 36 per Mb in *G. arboreum* chromosome A_A12 which is lower than in *G. raimondii* chromosome (53 per Mb of chromosome) [36]. Almost similar difference in gene density was reported between A12 and D12 chromosomes of *G. hirsutum* (29.4 vs 50 per Mb of chromosome) [8] and *G. barbadense* (33 vs 55.2 per Mb of chromosome), respectively [37].

### Collinear and syntenic relationship

**Fig. 2:**
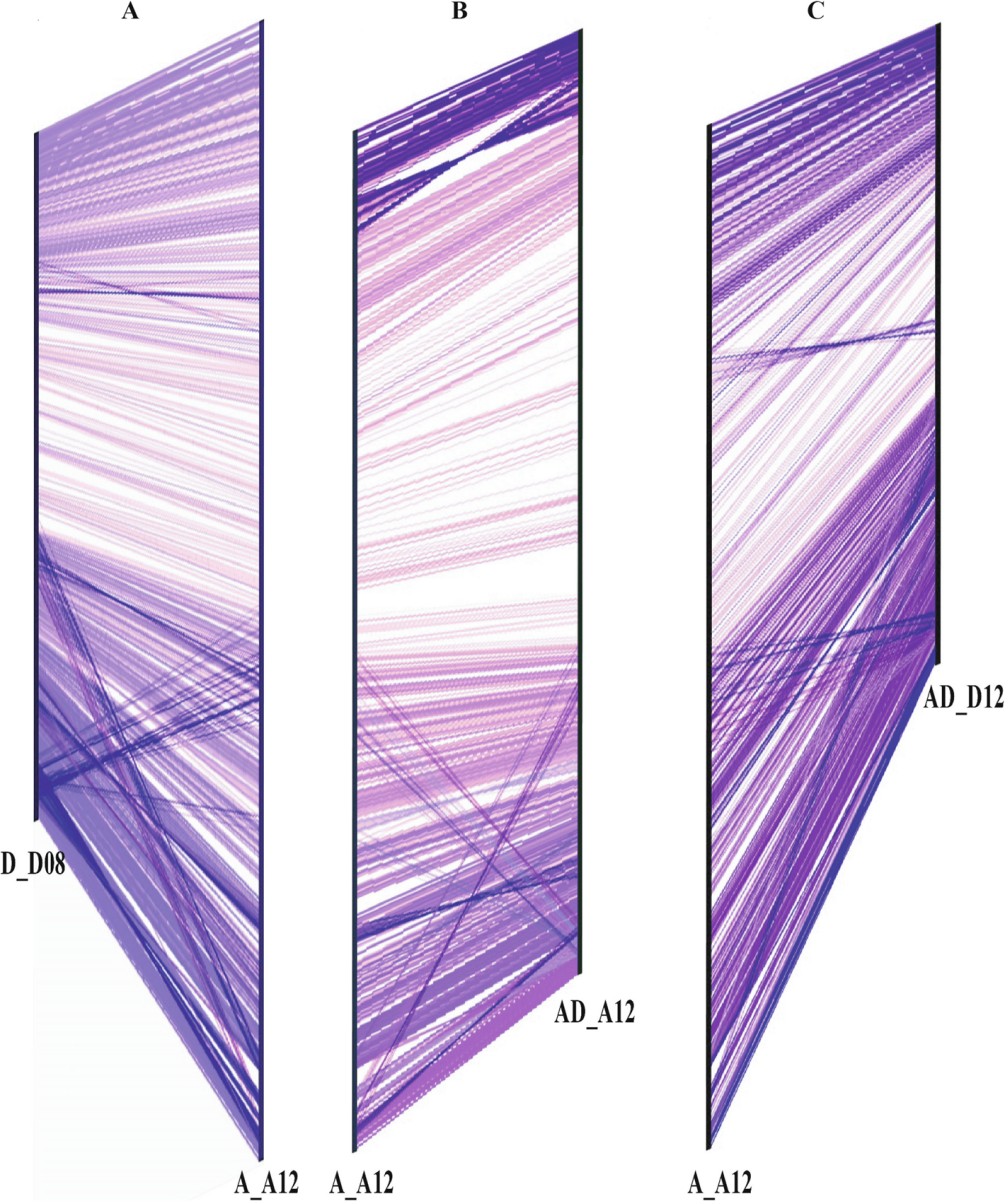
Syntenic relationship between corresponding homologous chromosomes of different *Gossypium* species. Syntenic relationship between homologous chromosomes 12 of; **a***G. raimondii* (D_D08) and *G. arboreum* (A_A12), **b***G. hirsutum* (AD_A12) and *G. arboreum* (A_A12), and **c***G. hirsutum* (AD_D12) and *G. arboreum* (A_A12). Syntenic blocks were required to match at least five genes per block after masking repeat regions. Good syntenic relationship was found when comparing the homologous chromosomes of *G. raimondii* (D_D08) and *G. hirsutum* (AD_A12 and AD_D12) with reassembled chromosome of *G. arboreum* (A_A12)

**Fig. 3:**
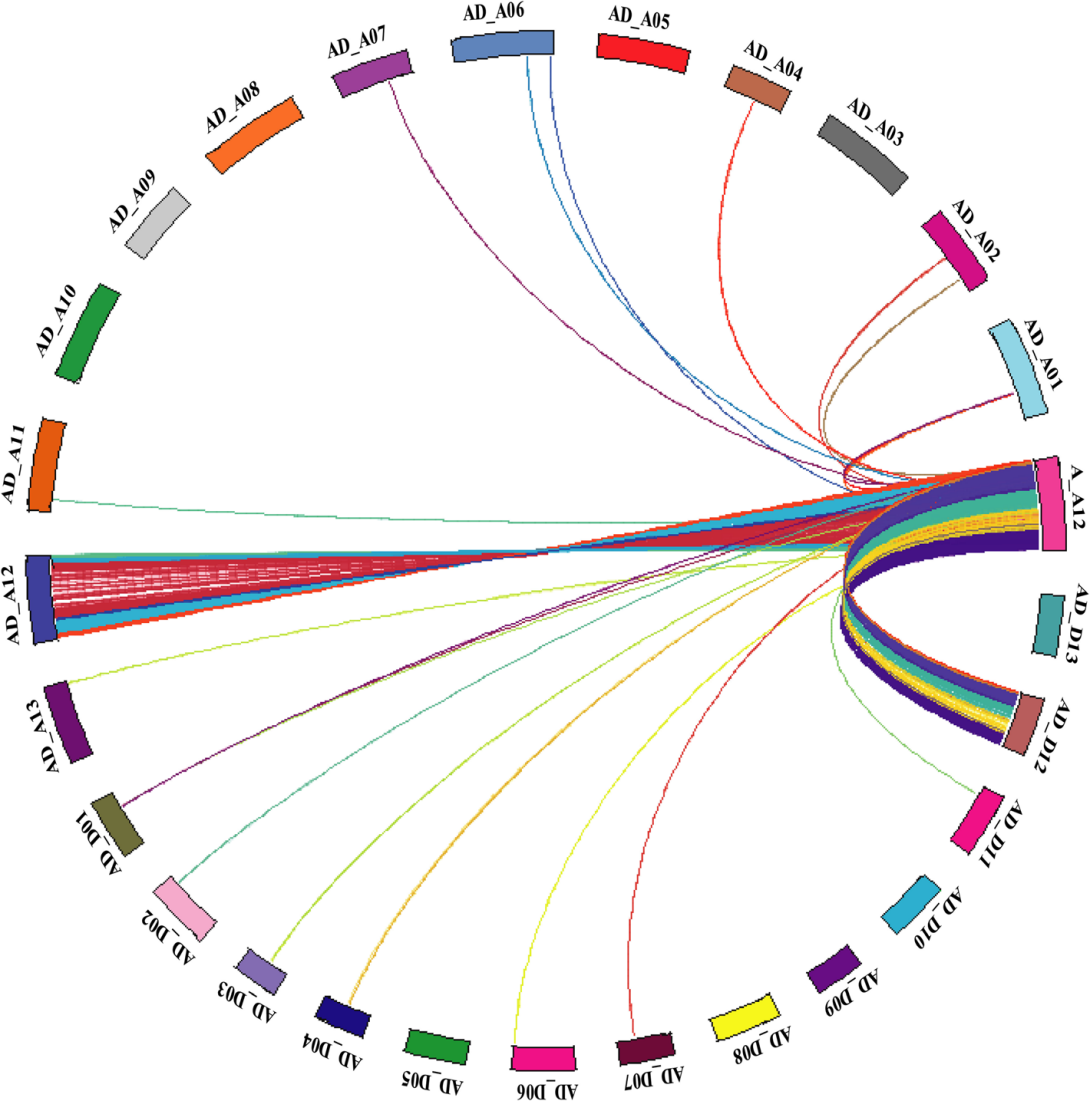
Collinearity of reassembled *G. arboreum* chromosome (A_A12) with 26 chromosomes of *G. hirsutum.* Collinear relationship of reassembled *G. arboreum* chromosome (A_A12) with 26 chromosomes of *G. hirsutum* was determined by MCScan. After masking the repeat regions, collinearity analysis of *G. arboreum* chromosome A_A12 was carried out with all 26 chromosomes of *G. hirsutum*. Results indicated good collinear relationship of reassembled *G. arboreum* chromosome A_A12 with its corresponding homologous chromosomes 12 (AD_A12 and AD_D12) of *G. hirsutum* as compare to others chromosomes. *G. arboreum* chromosome 12 was shown by ‘A_A12’ while, chromosomes belong to *A*_*t*_ and *D*_*t*_ sub-genomes of *G. hirsutum* were indicated by ‘AD_A’ and ‘AD_D’

**Fig. 4:**
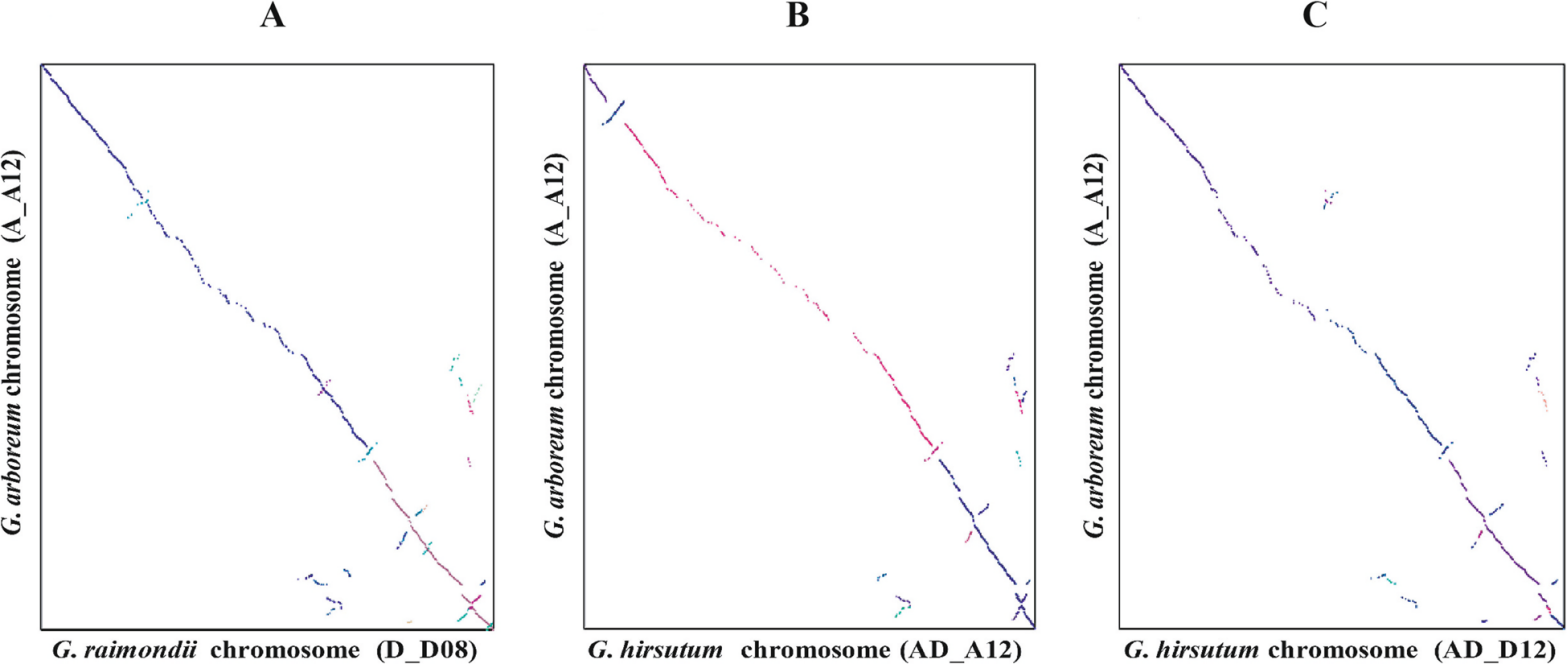
Dotplot representation between homologous chromosomes of different cotton species. A BLASTP search (with an E-value cutoff of 1 × 10^− 5^) was performed to identify orthologous genes. Afterwards, dotplots representation among homologous chromosomes of three cotton species was carried out by MCScan. **a***G. arboreum* chromosome A_A12 (Y-axis) vs *G. raimondii* chromosome D_D08 (X-axis), **b***G. arboreum* chromosome A_A12 (Y-axis) vs *G. hirsutum* chromosome AD_A12 (X-axis), and **c***G. arboreum* chromosome A_A12 (Y-axis) vs *G. hirsutum* chromosome AD_D12 (X-axis)

**Fig. 5:**
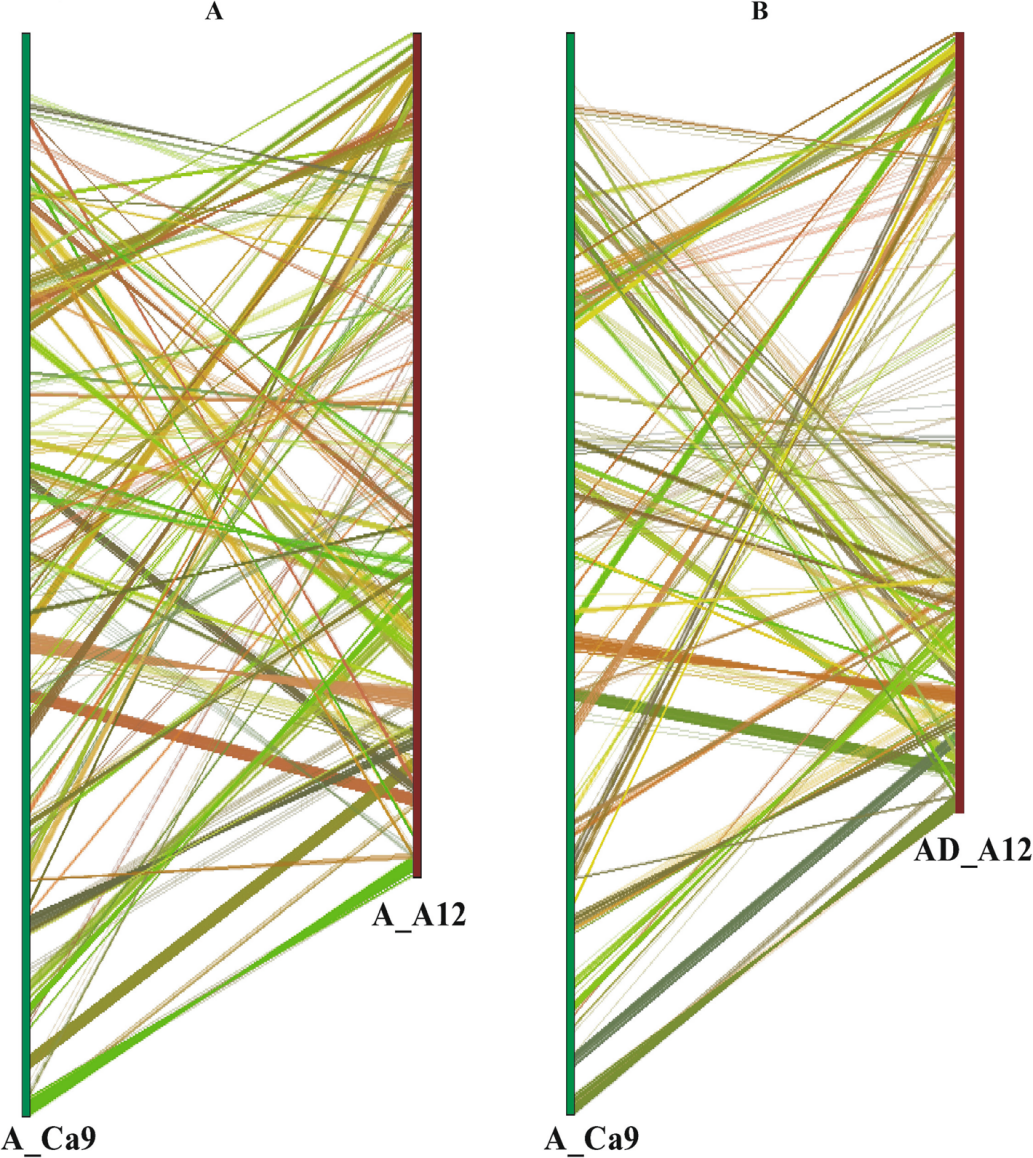
Syntenic relationship with previously assembled chromosome 12 of *G. arboreum* (A_Ca9)*.* Previously assembled chromosome 12 (A_Ca9) of *G. arboreum* was used to explore the syntenic relationship with **a** re-assembled *G. arboreum* chromosome A_A12 and, **b***G. hirsutum* chromosome AD_A12. Syntenic blocks were required to match at least five genes per block. Results indicated poor syntenic relationship of *G. arboreum* chromosome A_Ca9 with these two chromosomes

Comprehensive search of synteny and collinearity was carried out using BLASTP search comparing *G. arboreum* chromosome A_A12 with its corresponding homologous chromosomes of *G. raimondii* (D_D08) [36] and *G. hirsutum* (AD_A12 and AD_D12) [8]. Results indicated that the corresponding homologous chromosomes 12 of different *Gossypium* species possess a good syntenic relationship (Fig. 2a-c) such as 25 and 18 collinear blocks (with ≥5 genes per block) were aligned with *G. raimondii* (D_D08) and *G. hirsutum* (AD_A12) chromosomes (Additional File 2: Table S6), respectively. Overall gene order and collinearity was also highly conserved (Fig. 3 and Fig. 4a-c, Additional File 1: Fig. S3 and Fig. S4) between re-assembled *G. arboreum* chromosome A_A12 with its homologous chromosomes of *G. raimondii* [36] and *G. hirsutum* [8]. However, this collinearity was not apparent (Fig. 5a-b, Additional File 1: Fig. S5) with previously assembled *G. arboreum* chromosome (A_Ca9) [27], mainly due to; (i) mistakes in ordering of scaffolds (ii) many scaffolds belong to *G. arboreum* chromosome A_A12 were not present in it and, (iii) many scaffolds from other chromosomes were anchored and oriented in *G. arboreum* chromosome A_A12.

### Identification of orthologous gene pairs

We identified 2382 and 2603 orthologous gene pairs within homologous chromosomes (AD_A12 and AD_D12) of *G. hirsutum* and subsequent ancestral diploid A_A12 and D_D08 chromosomes (Additional File 2: Table S7). A total of 2485 ortholog pairs were identified between diploid A_A12 and D_D08 chromosomes.

### Gene loss

Gene order was generated among the homologous chromosomes 12 of three *Gossypium* species by quartet alignments in MCScan [38]. Flanking gene method has been used to find gene loss in the syntenic blocks. Homologous chromosomes of allotetraploid cotton have greater gene loss; 26 genes were lost from AD_A12 and 22 from AD_D12 chromosomes (Table 2). In contrast, 13 and 09 genes were absent from A_A12 and D_D08 chromosomes of *G. arboreum* and *G. raimondii*, respectively (Table 3).

**Table 2:** Gene loss in homologous chromosomes 12 of *G. hirsutum*

Genes loss in AD_A12 chromosome			Genes loss in AD_D12 chromosome		
D_D08	A_A12	AD_D12	D_D08	A_A12	AD_A12
Gorai.008G015200	Cotton_A_15792	Gh_D12G0137	Gorai.008G026700	Cotton_A_10793	Gh_A12G0236
Gorai.008G041500	Cotton_A_02090	Gh_D12G0372	Gorai.008G063700	Cotton_A_11364	Gh_A12G0558
Gorai.008G063000	Cotton_A_11373	Gh_D12G0567	Gorai.008G080200	Cotton_A_34337	Gh_A12G0688
Gorai.008G106800	Cotton_A_31201	Gh_D12G0942	Gorai.008G095800	Cotton_A_35255	Gh_A12G0798
Gorai.008G110900	Cotton_A_27718	Gh_D12G0984	Gorai.008G157500	Cotton_A_23027	Gh_A12G1304
Gorai.008G133700	Cotton_A_26243	Gh_D12G1202	Gorai.008G160900	Cotton_A_35616	Gh_A12G1336
Gorai.008G136900	Cotton_A_22647	Gh_D12G1233	Gorai.008G161100	Cotton_A_30134	Gh_A12G1338
Gorai.008G138200	Cotton_A_22060	Gh_D12G1246	Gorai.008G164600	Cotton_A_21032	Gh_A12G1366
Gorai.008G141000	Cotton_A_33185	Gh_D12G1271	Gorai.008G171300	Cotton_A_31070	Gh_A12G1433
Gorai.008G159100	Cotton_A_23046	Gh_D12G1444	Gorai.008G187700	Cotton_A_38211	Gh_A12G1570
Gorai.008G165500	Cotton_A_21019	Gh_D12G1498	Gorai.008G190100	Cotton_A_25801	Gh_A12G1593
Gorai.008G178300	Cotton_A_06177	Gh_D12G1616	Gorai.008G193900	Cotton_A_13403	Gh_A12G1616
Gorai.008G182200	Cotton_A_06137	Gh_D12G1649	Gorai.008G206100	Cotton_A_08046	Gh_A12G1715
Gorai.008G188300	Cotton_A_25782	Gh_D12G1706	Gorai.008G217000	Cotton_A_13589	Gh_A12G1810
Gorai.008G194300	Cotton_A_13398	Gh_D12G1760	Gorai.008G230800	Cotton_A_07177	Gh_A12G1938
Gorai.008G196900	Cotton_A_13365	Gh_D12G1787	Gorai.008G240500	Cotton_A_07085	Gh_A12G2029
Gorai.008G202800	Cotton_A_27500	Gh_D12G1844	Gorai.008G241600	Cotton_A_07074	Gh_A12G2040
Gorai.008G203500	Cotton_A_08073	Gh_D12G1852	Gorai.008G283400	Cotton_A_01373	Gh_A12G2388
Gorai.008G231000	Cotton_A_07174	Gh_D12G2120	Gorai.008G020900	Cotton_A_24594	Gh_A12G0175
Gorai.008G235800	Cotton_A_07128	Gh_D12G2164	Gorai.008G151100	Cotton_A_30237	Gh_A12G1241
Gorai.008G242300	Cotton_A_14421	Gh_D12G2224	Gorai.008G207500	Cotton_A_8032	Gh_A12G1729
Gorai.008G268800	Cotton_A_19242	Gh_D12G2414	Gorai.008G244100	Cotton_A_14443	Gh_A12G2062
Gorai.008G017400	Cotton_A_15816	Gh_D12G0157			
Gorai.008G077500	Cotton_A_31087	Gh_D12G0672			
Gorai.008G170600	Cotton_A_25559	Gh_D12G1546			
Gorai.008G194300	Cotton_A_13398	Gh_D12G1760			

A_A12, *G. arboreum* chromosome; D_D08, *G. raimondii* chromosome; AD_A12 & AD_D12, *G. hirsutum* chromosomes

**Table 3:** Gene loss in homologous chromosomes 12 of *G. arboreum* and *G. raimondii*

Genes loss in A_A12 chromosome			Genes loss in D_D08 chromosome		
AD_D12	AD_A12	D_D08	AD_D12	AD_A12	A_A12
Gh_D12G0154	Gh_A12G0141	Gorai.008G016900	Gh_D12G0046	Gh_A12G0031	Cotton_A_21998
Gh_D12G1069	Gh_A12G0957	Gorai.008G119400	Gh_D12G0145	Gh_A12G0131	Cotton_A_15801
Gh_D12G1172	Gh_A12G1052	Gorai.008G130700	Gh_D12G0571	Gh_A12G0555	Cotton_A_11368
Gh_D12G1313	Gh_A12G1191	Gorai.008G145300	Gh_D12G0937	Gh_A12G0857	Cotton_A_29573
Gh_D12G1414	Gh_A12G1292	Gorai.008G156000	Gh_D12G1073	Gh_A12G0961	Cotton_A_20925
Gh_D12G1862	Gh_A12G1699	Gorai.008G204500	Gh_D12G1353	Gh_A12G1228	Cotton_A_14576
Gh_D12G2015	Gh_A12G1845	Gorai.008G220400	Gh_D12G1992	Gh_A12G1821	Cotton_A_13578
Gh_D12G2032	Gh_A12G1861	Gorai.008G222300	Gh_D12G2303	Gh_A12G2123	Cotton_A_23201
Gh_D12G2315	Gh_A12G2135	Gorai.008G254600	Gh_D12G2444	Gh_A12G2310	Cotton_A_01291
Gh_D12G2573	Gh_A12G2447	Gorai.008G291600			
Gh_D12G2634	Gh_A12G2507	Gorai.008G297900			
Gh_D12G2440	Gh_A12G2304	Gorai.008G275000			
Gh_D12G0980	Gh_A12G0894	Gorai.008G110500			

A_A12, *G. arboreum* chromosome; D_D08, *G. raimondii* chromosome; AD_A12 & AD_D12, *G. hirsutum* chromosomes

### Identification and mapping of transcription factor (TF) related genes

**Fig. 6:**
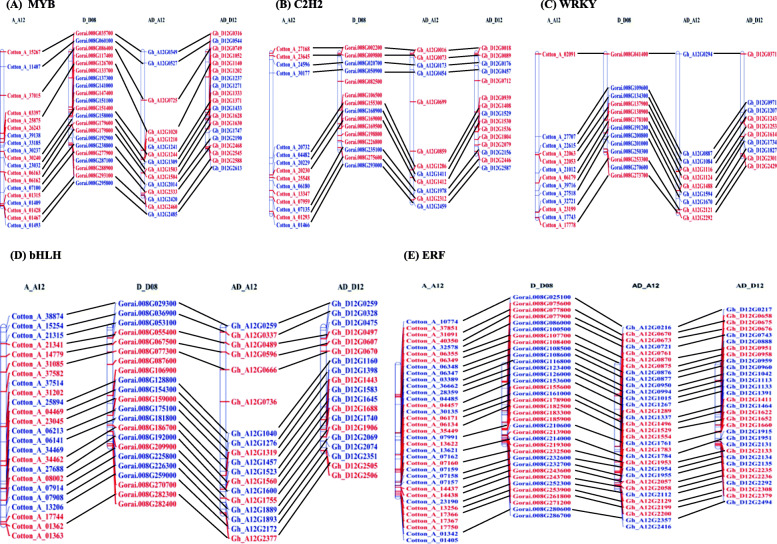
Chromosomal mapping of the TF-related genes on homologous chromosome 12 of three cotton species. Physical mapping of five major TF-related family members including (**a**) *MYB*, (**b**) *C2H2*, (**c**) *WRKY*, (**d**) *bHLH*, (**e**) *ERF* was performed in homologous chromosome 12 of *G. arboreum* (A_A12), *G. raimondii* (D_D08) and *G. hirsutum* (AD_A12 and AD_D12). Genes in the positive and negative strands were represented by blue and red colors, while lines signified the collinear genes

Firstly, we generated an updated list of putative TF related genes of *G. arboreum* chromosome A_A12 using PlantTFDB [39]. This led to the identification of 266 putative members from 40 TF families, representing 8% of the protein-coding genes (Additional File 2: Table S8). There was more enrichment of *ERF* (35) related genes on chromosome A_A12 followed by *bHLH* (24), *MYB* (19), *C2H2* (15) and *WRKY* (13). We also identified TF members of these five major families (*ERF, bHLH, MYB, C2H2* and *WRKY*) in homologous chromosomes 12 of *G. raimondii* and *G. hirsutum* (Additional File 2: Table S9) to observe the influence of allopolyploidy on these genes. Comparative physical mapping of these genes on homologous chromosomes 12 of diploid and tetraploid cotton species revealed good collinear relationships among most of the TF-related genes (Fig. 6a-e). In particular, the chromosomal distribution of TF members in AD_A12 and AD_D12 chromosomes were more similar to their diploid progenitor’s chromosomes (A_A12 and D_D08). Moreover, TF encoding genes were not evenly distributed within the chromosomes. In general, the central region of chromosomes contained less number of TF-related genes, while comparatively high densities of TF members were found in bottom section of chromosomes.

## Discussion

Chromosome-scale assemblies of sequenced plant genomes facilitated the discovery of important features of genome evolution. However, a consistent method for chromosome assembling from NGS data continues to present a serious constraint. Cultivated *G. arboreum* is important diploid cotton specie that contains important traits such as resistance to biotic and abiotic stresses [40, 41]. Previously, draft genome of *G. arboreum* has been sequenced and assembled [27] using 193.6 Gb of high-quality sequence reads. However, it contained several errors in ordering and orienting of scaffolds into pseudo-molecules [8, 30]. To address this problem, we re-constructed *G. arboreum* chromosome A_A12 by combining genetic mapping and reference assisted approaches. Initially, a high density genetic map of *G. arboreum* was constructed using 3735 good quality SNP markers from previous study [27], consisted of 3544 SNP loci and spanned 1599.8 cM in 13 linkage groups. Subsequently, linkage group belong to *G. arboreum* chromosome A_A12 was proceed for reassembling using reference assisted approach as it contains important genes for different traits [32–34], and do not contain any translocation [8, 35]. Final assembly of *G. arboreum* chromosome A_A12 comprised of 144 scaffolds and spanned 94.64 Mb length, which is almost twice the size (57.13 Mb) of its homologous chromosome (D_D08) of *G. raimondii* [36]. These results were consistent with chromosome size difference between the homologous chromosome 12 of *At* (87.4 Mb) and *Dt* (59.1 Mb) subgenome of *G. hirsutum* [8]. Similarly, tetraploid genome of *G. barbadense* [37] contained A12 and D12 chromosomes of the103.3 Mb and 58.2 Mb, respectively.

Further, both *G. arboreum* and *G. raimondii* chromosomes (A_A12 and D_D08) contained 3361 and 2990 genes, resulted lower gene density (36 vs 53 per Mb of chromosome) in A_A12 chromosome than D_D08 [36]. Similar difference in gene density was observed between the A12 and D12 chromosomes of *G. hirsutum* [8] and *G. barbadense* [37]. This lower gene density in chromosome A_A12 than D_D08 is mainly due to the presence of more repetitive elements. Previously, several studies also reported that larger genome size of *G. arboreum* relative to *G. raimondii* was mainly due to the presence of repetitive elements [42, 43]. Additionally, *G. arboreum* genome contained [27] high percentage of transposable elements as compared to *G. raimondii* [7, 36].

Polyploidization is often followed by whole genome duplication that is illustrated by genome reorganization and immense gene loss [44–46]. This process has been observed in different plants i.e. wheat [47], *Brassica* [48] and maize [49]. Though, some other plants including *Arabidopsis* [50] and cotton [51] do not illustrate various changes in their genome sequences. In current study, synteny and collinearity, whole chromosomal alignment and homologous gene dotplotting showed highly conserved syntenic and collinear relationship among homologous chromosomes of *G. hirsutum*, *G. raimondii* and reassembled *G. arboreum* chromosome, depicting preservation of very similar genomic structure since their divergence [52, 53]. Previous studies also reported highly conserved collinear relationship among different cotton species, which is also consistent to our results [8, 54]. This is possibly because actual progenitors which may form stable cultivated allotetraploid were lost or unstable tetraploid was eliminated by natural selection during early generations. However, this synteny was not apparent with previously assembled chromosome of *G. arboreum* (A_Ca9) [27]. In addition, homologous gene dotplotting with *G. arboreum* chromosome A_Ca9 also showed unobvious collinear relationship, confirming various mistakes in ordering and anchoring of scaffolds. Previous report [8] also showed unobvious collinearity between the homologous chromosomes of *G. hirsutum* and *G. arboreum*, which was consistent to our result.

Differential gene loss is an important factor during genome evolution which affects synteny between corresponding regions of different chromosomes [55–57], and can lead to immediate loss of gene function. In current study, we found a higher rate of gene loss in homologous chromosomes of tetraploid (AD_A12 and AD_D12) than diploid (A_A12 and D_D08) cotton. These results were consistent with the previous reports [8, 28], suggesting gene loss is probably an enduring process in chromosomal evolution of tetraploid cotton.

Transcription factors play a significant role in plant growth and development, secondary metabolism, organ morphogenesis and resistance against different stresses in cotton [58–60]. Several previous reports computed genome-wide analysis of TF-related genes in different cotton species and compared their physical location on different chromosomes [61–64]. In current study, distribution of TF-related genes showed that homologous chromosomes of *G. raimondii* (D_D08) and *G. arboreum* (A_A12) contained almost similar number of TF genes with minimum deviation, and they had good collinear relationship with each other. For Instance, 13 *WRKY* genes were identified on each of re-constructed *G. arboreum* A_A12 and *G. raimondii* D_D08 chromosomes with high collinearity. Recent study also reported highly conserved collinearity among TF-related genes of four *Gossypium* species [65]. In contrast, another study using previously assembled *G. arboreum* genome [27] identified different number of *WRKY* genes and their unobvious collinearity in *G. arboreum* and *G. raimondii* chromosomes 12, respectively [63]. Furthermore, distribution of TF encoding genes was not even within the corresponding homologous chromosome of three cotton species which is likely due to sequence exchange through recombination mispairing [66].

## Conclusion

In conclusion, we generated an improved reassembly of *G. arboreum* chromosome A_A12 using NGS data of previous study [27] by combining genetic mapping and reference assisted approaches. This study provides an initial more accurate strategy for correcting mis-assemblies in sequenced genome of *G. arboreum* which can also be applied to improve chromosome-scale assemblies of large and complex plant genomes without having good genetic or physical maps.

## Methods

### Genomes and markers data

Sequenced genome data of *G. arboreum* [27] including scaffolds, predicted annotated genes and genotypic data of 24,569 SNP markers as well as scaffolds data of *G. raimondii* [7] was obtained from Institute of Cotton Research, Chinese Academy of Agricultural Sciences, Anyang, China. Chromosomal and genes annotation data of *G. hirsutum* [8] and *G. raimondii* [36] was downloaded from the CottonFGD (https://cottonfgd.org/). Meanwhile, sequence data of previous mapped markers of *G. hirsutum* and *G. raimondii* for each chromosome was downloaded from COTTONGEN (https://www.cottongen.org/find/markers).

### SNP markers selection

Markers data of 24,569 SNPs [27] was filtered out to obtain good quality linkage map of *G. arboreum*. Firstly, Chi-square test was executed to find markers diverging from Mendelian segregation patterns. Markers were excluded from analysis when they displayed very significant distortion (*P* < 0.01) from expected segregation ratio, also when they had more than 30% missing genotypic data. We identified markers with more than 95% similarity, and only one such marker was used for linkage map analysis.

### Genetic map construction

Linkage groups were constructed by JoinMap 4.0 [67] using F_2_ generation from previous study [27]. Markers were allocated to linkage groups by independence logarithm of odds (LOD) of 2.5–50.0 with a step of 1.0. Linkage groups were generated using LOD thresholds of 6.0 and maximum recombination thresholds of 0.4. We used a maximum likelihood mapping algorithm for calculation efficiency of marker order [68] if linkage group contained more than 500 markers. However, the scope of corresponding linkage groups (3000–6000 cM) exceeded JoinMap 4.0. Therefore, linkage length was divided by 100 for the presentation of genetic map [69]. In other linkage groups having less than 500 markers, a linear regression algorithm and the Kosambi mapping function [70] was used to convert recombination frequencies into centiMorgan (cM) map distances. Final linkage map was drawn using Mapchart 2.2 [71].

### Assign chromosomes names to linkage groups

To assign chromosomes names to each linkage group, sequence data of mapped markers for each chromosome of *G. hirsutum* and *G. raimondii* was obtained from COTTONGEN (https://www.cottongen.org/find/markers). Then a BLAST search was made using the marker sequence data of *G. hirsutum* and *G. raimondii* as a query and *G. arboreum* scaffolds corresponding to SNP markers of each linkage group as a database.

### Initial alignment of *G. arboreum* scaffolds

All scaffolds belonging to 189 SNP markers of *G. arboreum* chromosome A_A12 were pairwise aligned with the *G. raimondii* scaffolds [7] by BLAST-Like Alignment Tool (BLAT). The resulted alignments were required to have score values showing the length and similarity of aligned regions, while only best BLAT hit was counted from the alignments. Afterward, each of the pairwise alignment was validated by anchoring the protein coding genes of *G. raimondii* scaffolds [7] within *G. arboreum* scaffolds by BLASTN. If a gap between two coordinated scaffolds was > 100 kb then the corresponding region of D scaffolds was extracted to align it with the scaffolds of *G. arboreum* (as a database) by BLAT followed by gene wise BLASTN. This step is repeated until maximum number of *G. arboreum* scaffolds were aligned with the *G. raimondii* scaffolds [7].

### Final alignment of *G. arboreum* scaffolds

Next, all *G. raimondii* and *G. arboreum* scaffolds [7] obtained by initial alignment were separately pair-wise aligned with another version of *G. raimondii* chromosome (D_D08) [36] via BLAT and gene wise BLASTN. Unlocated and unplaced scaffolds of *G. arboreum* were excluded from the assembly. Again, if a gap between two coordinated scaffolds was more than 100 kb, the corresponding nucleotide sequence of *G. raimondii* chromosome (D_D08) was extracted and used as a query to align it with *G. arboreum* scaffolds by BLAT and BLASTN. Eventually, all resulted scaffolds were further confirmed by arranging them on the homologous chromosome (AD_A12) of *G. hirsutum* [8].

### Correction of assembly using genetic map and syntenic relationship

The linkage map of *G. arboreum* chromosome A_A12 and its synteny with the homologous chromosome of *G. raimondii* [36] and *G. hirsutum* [8] was used to find false joins within the scaffolds and to anchor the scaffolds into chromosome. Scaffolds were broken if they enclosed a false join based on genetic map and syntenic relationship. Then, corrected scaffolds were arranged to generate chromosome A_A12 of *G. arboreum*.

### Gene contents of *G. arboreum* chromosome A_A12

An AGP (a golden path) file that records the position of protein-coding genes for each scaffold of *G. arboreum* [27] was obtained from Institute of Cotton Research, Chinese Academy of Agricultural Sciences, Anyang, China. We generated an updated list of genes and proteins for re-assembled *G. arboreum* chromosome A_A12 by arranging the genes and proteins of each scaffolds in their respective order. Putative functional description of all genes was explored by CottonFGD (https://cottonfgd.org/search/).

### Syntenic and collinear analysis

Syntenic blocks between corresponding homologous chromosomes of *G. arboreum* (A_A12)*, G. hirsutum* (AD_A12 and AD_D12) [8] and *G. raimondii* (D_D08) [36] were identified by MCScan [38] with default parameters. After removing multiple matches and tandem duplications, syntenic blocks having more than five gene pairs were identified.

### Identification of orthologous gene sets

All protein sequences of corresponding homologous chromosomes 12 of each cotton species (*G. arboreum*, *G. raimondii* and *G. hirsutum*) were compared by BLASTP (e*-*value < 1 × 10^− 5^). Genes were classified into ortholog clusters with OrthoMCL against OrthoMCL database proteins [72]. Multiple sequence alignment of *G. arboreum*, *G. raimondii* and *G. hirsutum* protein coding sequences was performed with ClustalW [73]. Based on the orthologous gene sets between homologous chromosomes of *G. arboreum* (A_A12)*, G. raimondii* (D_D08) [36], and two sub-genomes of *G. hirsutum* (AD_A12 and AD_D12) [8], synonymous and non-synonymous substitutions per site among three cotton species were calculated by Synonymous Non-synonymous Analysis Program (SNAP) [74].

### Gene loss

Gene-loss events were depicted using flanking gene method from the synteny table generated by MCScan [38]. For instance, given flanking genes X, Y and Z in order, if gene Y is present in the corresponding homologous chromosomes 12 of three *Gossypium* genomes, but missed in chromosome of other one genome, then gene Y is referred as a lost gene. However, both X and Z genes are essentially to be present in homologous chromosome (A_A12, D_D08, AD_A12 and AD_D12) of all four *Gossypium* genomes.

### Identification and mapping of transcription factor related genes

Transcription factor (TF) related genes were identified by searching all protein sequences of re-assembled *G. arboreum* chromosome A_A12 using Plant Transcription Factor Database, PlantTFDB [39]. Afterwards, only top five putative TF-related genes including *ERF, bHLH, MYB, C2H2* and *WRKY* were used for further analysis. The Hidden Markov Model (HMM) profiles of gene domains were obtained from Pfam [75] for gene family identification. HMMER 3.0 [76] search was used to confirm the putative TF-related genes in homologous chromosomes 12 of *G. arboreum, G. raimondii* and *G. hirsutum.* Chromosomal position of all TF-related genes was resolved by BLASTN searches against chromosomes of *G. arboreum* (A_12), *G. raimondii* (D_D08) [36] and *G. hirsutum* (AD_A12 and AD_D12) [8]. All TF-related genes were mapped on the chromosomes using the Mapchart 2.2 [71].

## Supplementary information

**Additional file 1: Fig. S1** Genetic map of *G. arboreum* genome. **Fig. S2** Arrangement of *G. arboreum* scaffolds within reassembled *G. arboreum* chromosome 12 (A_A12). **Fig. S3** Collinearity among homologous chromosomes 12 of three cotton species. **Fig. S4** Alignments of reassembled *G. arboreum* chromosome A_A12 with the whole genome of *G. hirsutum.***Fig. S5** Dotplot representation with the previously assembled *G. arboreum* chromosome.

**Additional file 2 Table S1** Genetic map construction of *G. arboreum.***Table S2** Chromosomes names assignment to each linkage group with respect to previous mapped markers of *G. raimondii.***Table S3** Chromosomes names assignment to each linkage group with respect to previous mapped markers of *G. hirsutum.***Table S4** Chromosomes names assignment to each linkage group with respect to previous mapped markers of *G. arboreum.***Table S5** Statistics for misassembled scaffolds. **Table S6** Collinear blocks among the homologous chromosome 12 of different cotton species. **Table S7** Orthologous gene pairs between homologous chromosomes 12 of different cotton species. **Table S8** TF-related genes in reassembled *G. arboreum* chromosome A_A12. **Table S9** TF-related genes on homologous chromosome 12 of three cotton species. (XLS 1436 kb)

## Data Availability

The sequence data of re-constructed *G. arboreum* chromosome 12 has been deposited at the NCBI Genbank under the accession number CP053561. The other data sets generated in this study are included within the article and supplementary files.

## Abbreviations

A_A12: Re-assembled *G. arboreum* chromosome 12
A_Ca9: Chromosome 12 of *G. arboreum* [27]
D_D08: Chromosome 12 of *G. raimondii* [36]
AD_A12: Chromosome 12 for At subgenome of *G. hirsutum* [8]
AD_D12: Chromosome 12 for Dt subgenome of *G. hirsutum* [8]
NGS: Next-generation sequencing
LRS: Long-read sequencing
cM: Centi-Morgan
LOD: Logarithm of the odds
SNP: Single nucleotide polymorphism
BLAST: Basic local alignment search tool
BLAT: BLAST-Like alignment tool
HMM: Hidden markov model
N50: 50% of the assembled nucleotides within scaffolds
TF: Transcription factor
