# Supplementary material for: Improved reconstruction and comparative analysis of chromosome 12 to rectify Mis-assemblies in Gossypium arboreum
Source: BMC Genomics. 2020 Jul 8;21:470. doi: 10.1186/s12864-020-06814-5 (PMC7346634; doi:10.1186/s12864-020-06814-5)
Supplement: Supplementary file 1 — Additional file 1: Fig. S1 Genetic map of G. arboreum genome. Fig. S2 Arrangement of G. arboreum scaffolds within reassembled G. arboreum chromosome 12 (A_A12). Fig. S3 Collinearity among homologous chromosomes 12 of three cotton species. Fig. S4 Alignments of reassembled G. arboreum chromosome A_A12 with the whole genome of G. hirsutum.Fig. S5 Dotplot representation with the previously assembled G. arboreum chromosome. [file 12864_2020_6814_MOESM1_ESM.docx]

**Improved Reconstruction and Comparative Analysis of Chromosome 12 to Rectify Mis-assemblies in *Gossypium arboreum***

Javaria Ashraf^1,2^, Dongyun Zuo^1,3^, Hailiang Cheng^1,3^, Waqas Malik^2^, Qiaolian Wang^1,3^, Youping Zhang^1,3^, Muhammad Ali Abid^2^, Qiuhong Yang^4^, Xiaoxu Feng^1,3^, John Z. Yu^5^ and Guoli Song^1,3*^

^*^ Correspondence: <sglzms@163.com>

^1^ Institute of Cotton Research, Chinese Academy of Agricultural Sciences, Anyang 455000, China

^2^ Genomics Lab, Department of Plant Breeding and Genetics, Faculty of Agricultural Sciences and Technology, Bahauddin Zakariya University, Multan, Punjab 60000, Pakistan

^3^ Zhengzhou Research Base, State Key Laboratory of Cotton Biology, Zhengzhou University, Zhengzhou 450001, China

^4^College of Life Sciences, Tarim University, Alar 843300, China

^5^ Crop Germplasm Research Unit, Southern Plains Agricultural Research Center, US Department of Agriculture–Agricultural Research Service (USDA-ARS), College Station, Texas 77845, USA.

^*^ Correspondence should be addressed to G.S. (Tel 86 372 2562377; fax 86 372 2562256; email: sglzms@163.com)


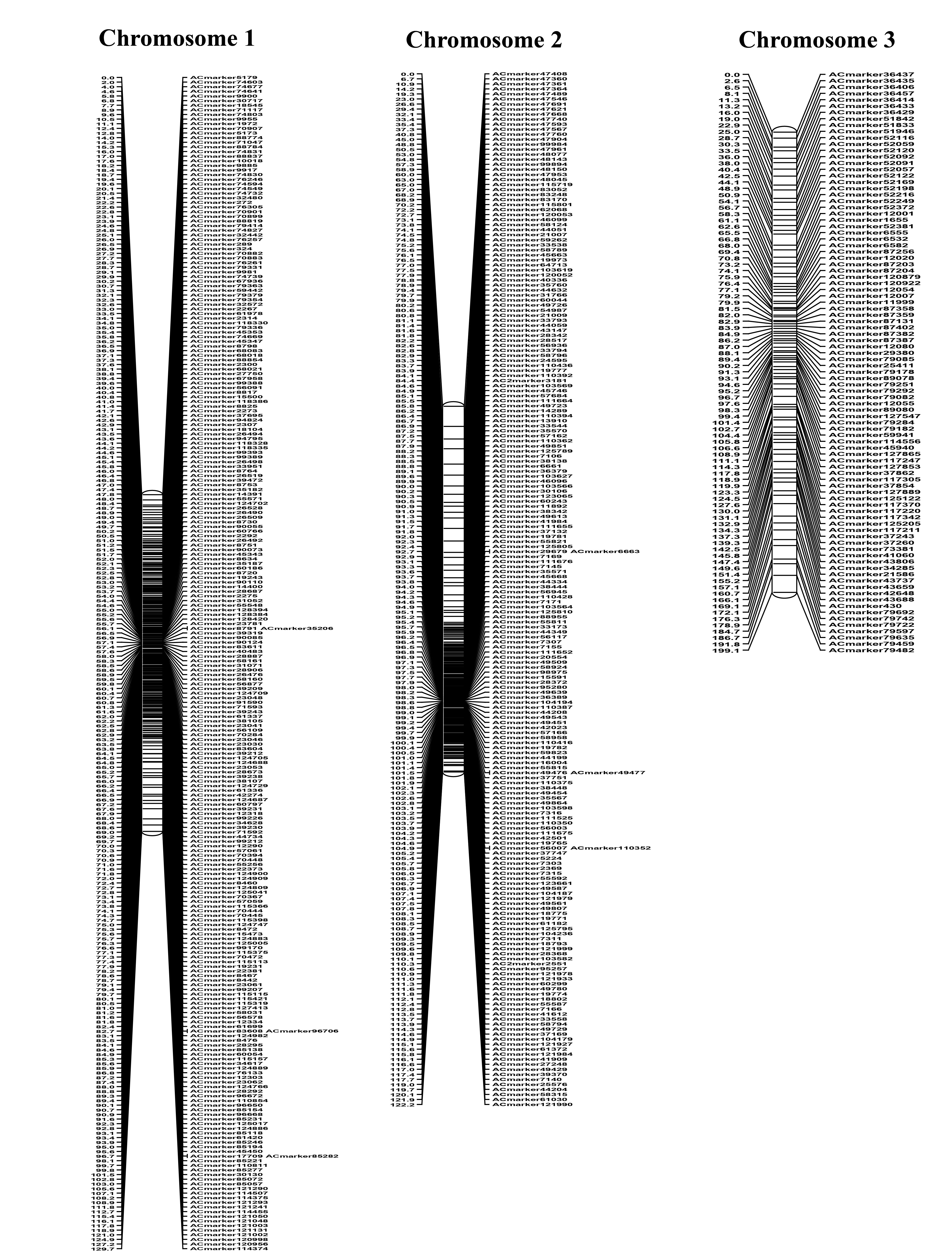


**Fig. S1 Genetic map of *G. arboreum* genome.**

Genetic distances were listed on the left side of each group and respective marker IDs on the right side.

**
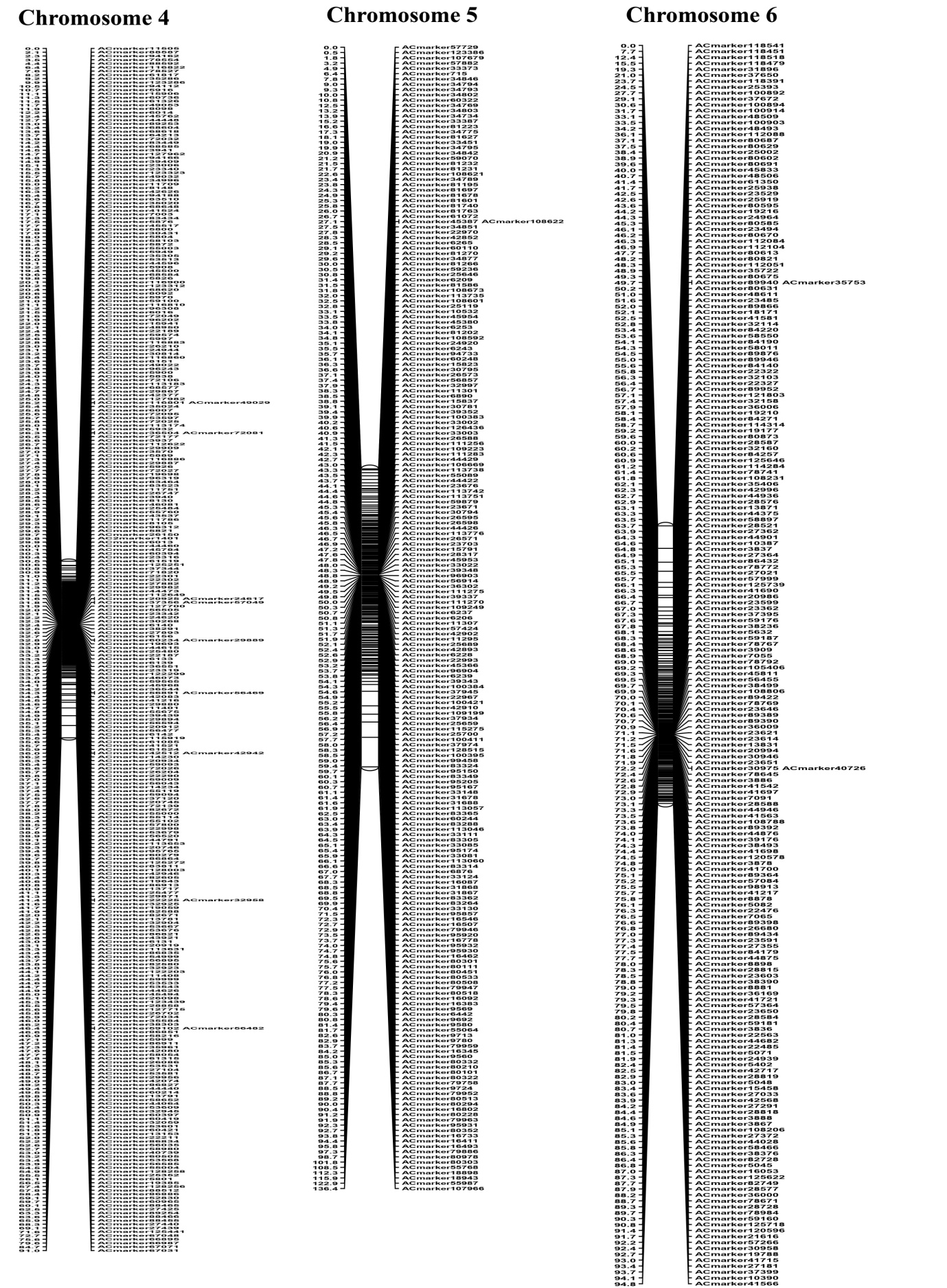
**

**Fig. S1 Genetic map of *G. arboreum* genome (Continued)**

Genetic distances were listed on the left side of each group and respective marker IDs on the right side.

**
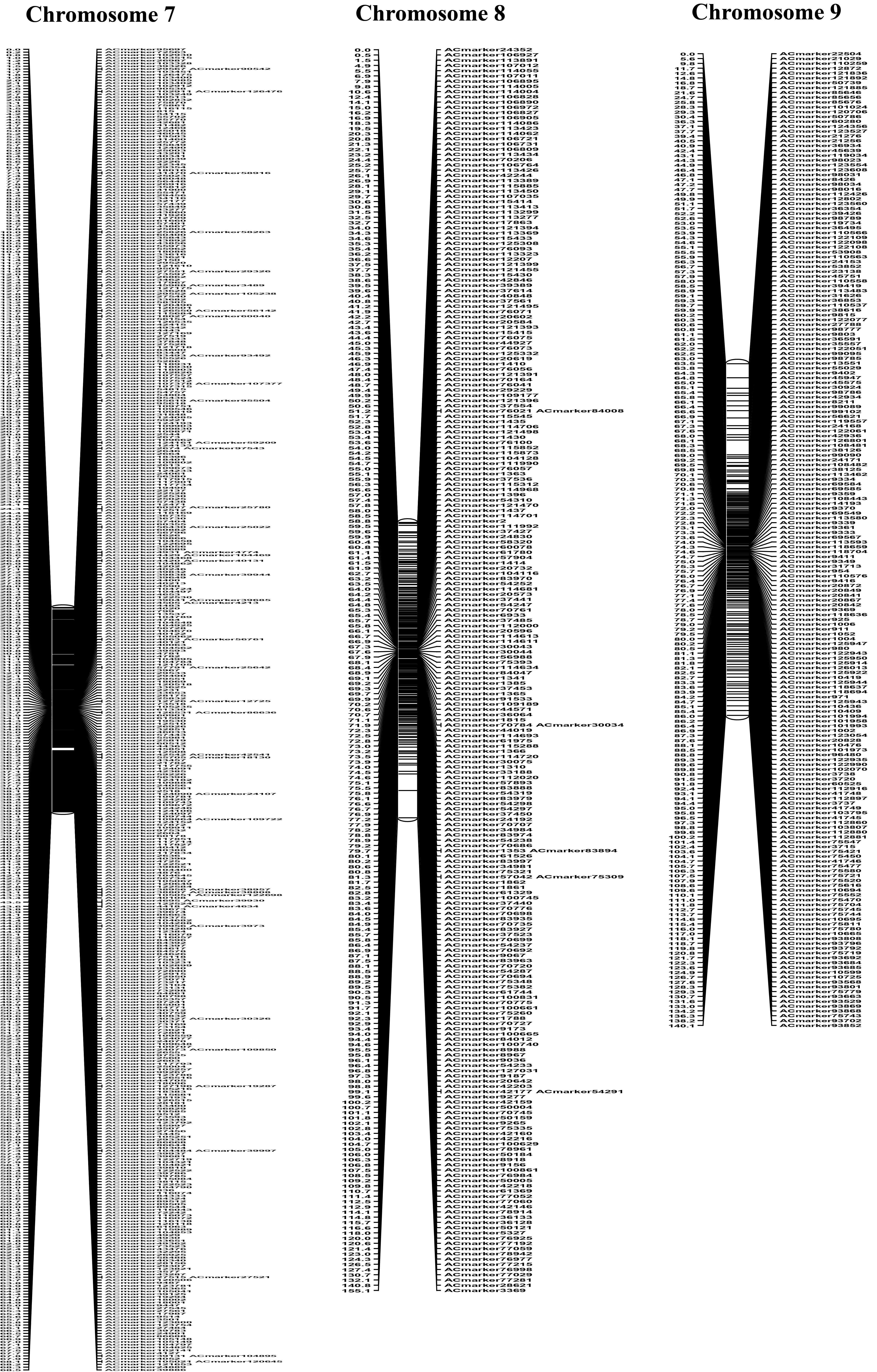
**

**Fig. S1 Genetic map of *G. arboreum* genome (Continued)**

Genetic distances were listed on the left side of each group and respective marker IDs on the right side.

**
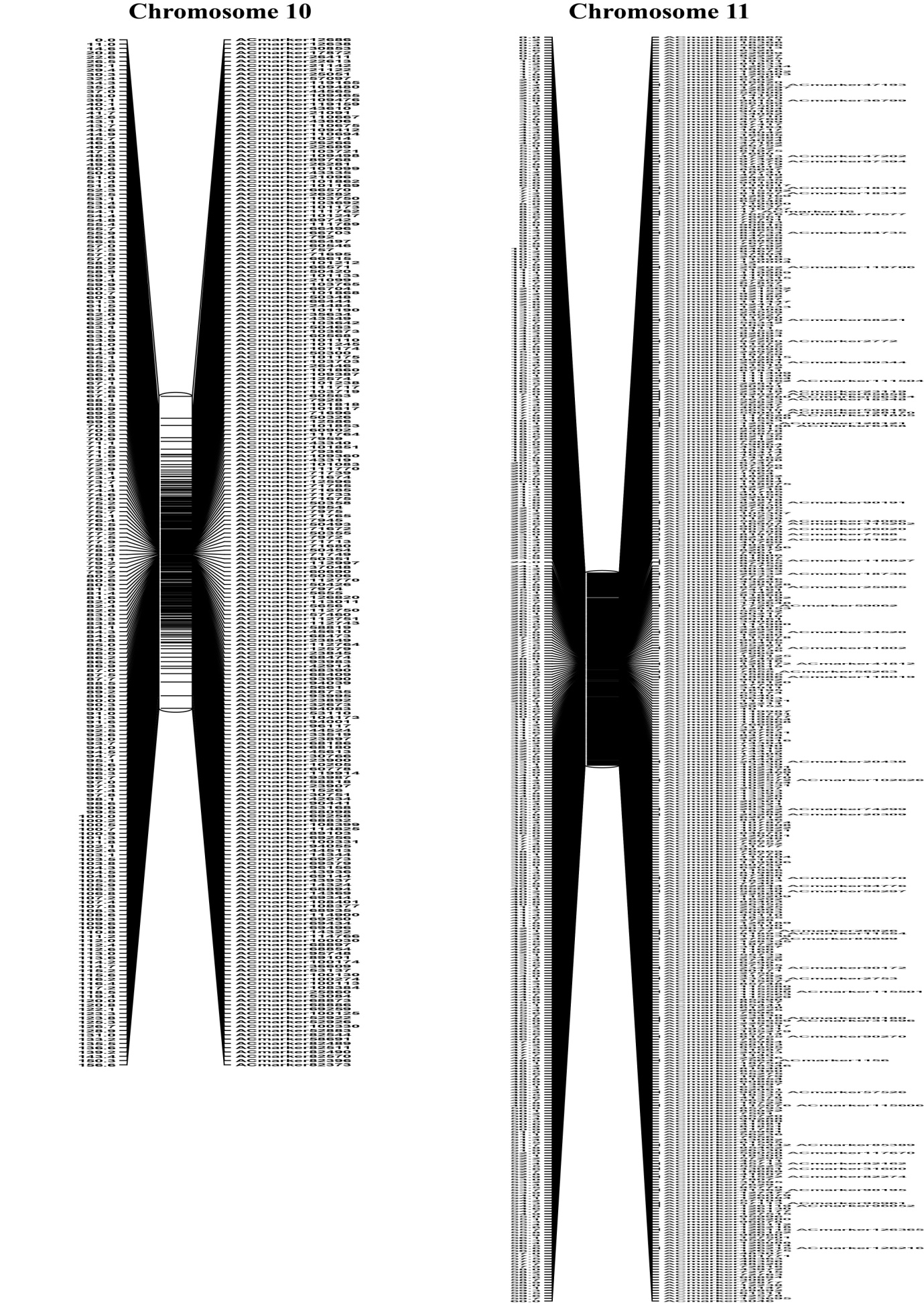
**

**Fig. S1 Genetic map of *G. arboreum* genome (Continued)**

Genetic distances were listed on the left side of each group and respective marker IDs on the right side.

**
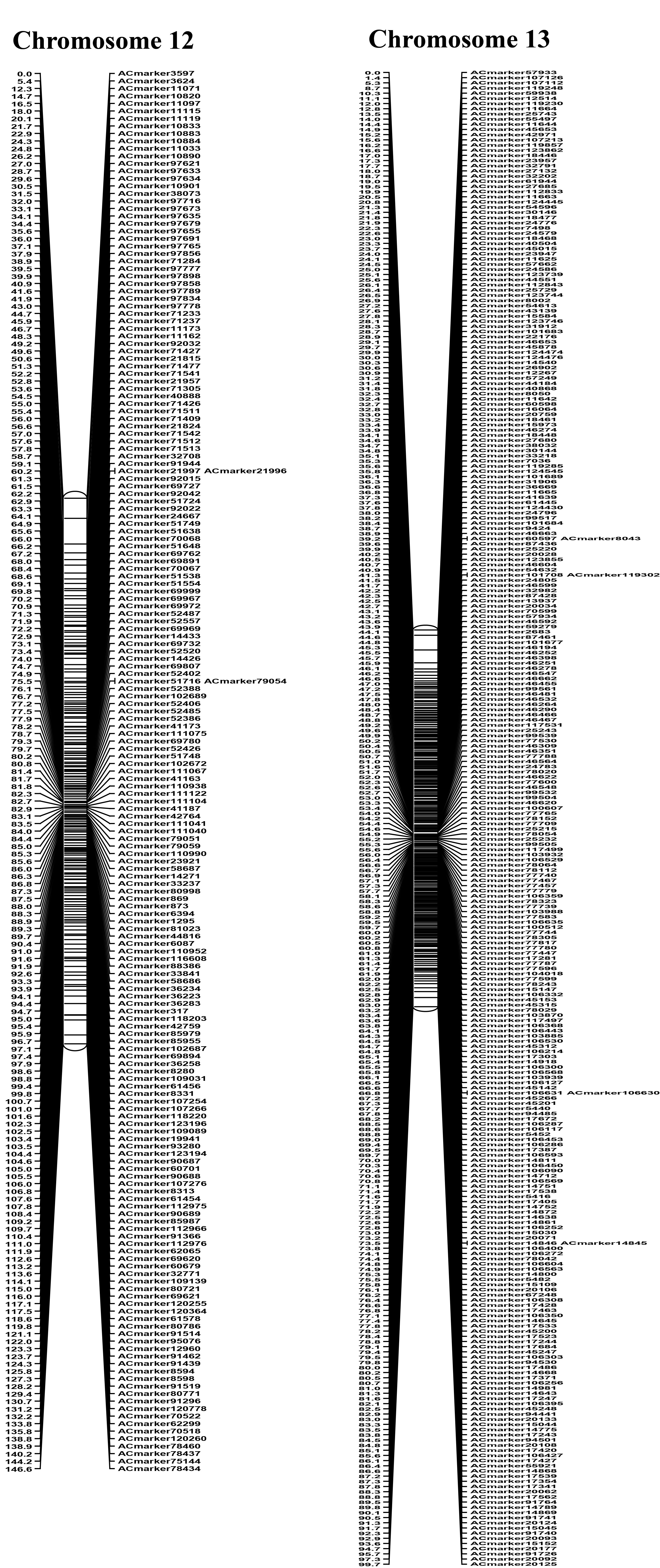
**

**Fig. Genetic map of *G. arboreum* genome (Continued)**

Genetic distances were listed on the left side of each group and respective marker IDs on the right side.

**
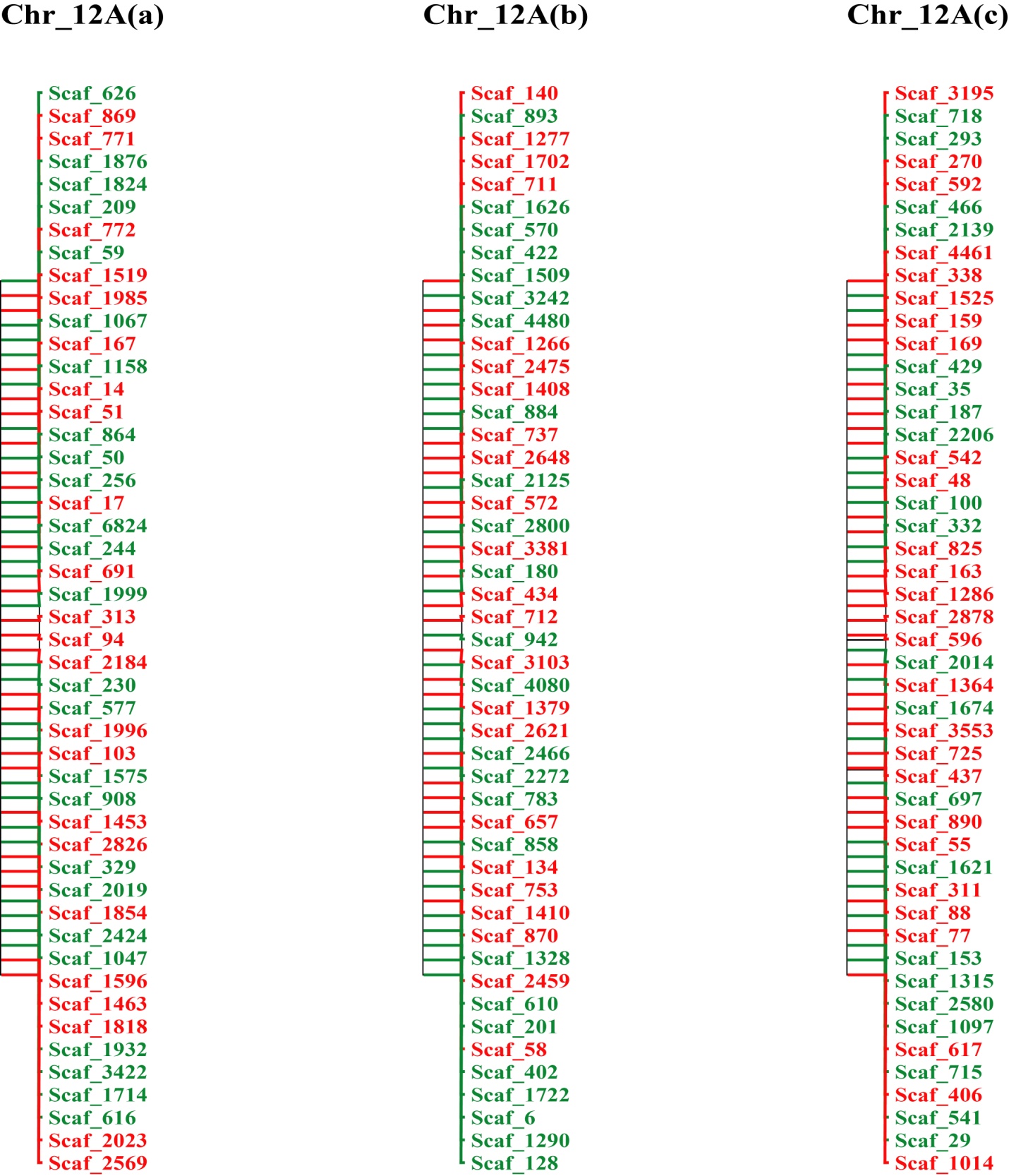
**

**Fig. S2 Arrangement of *G. arboreum* scaffolds within re-assembled *G. arboreum* chromosome 12 (A_A12).**

Final assembly of *G. arboreum* chromosome 12 (A_A12) comprised of 144 scaffolds which is generated by combining genetic mapping and reference assisted approaches. Scaffolds on positive and negative strands were represented by green and red colors, respectively.


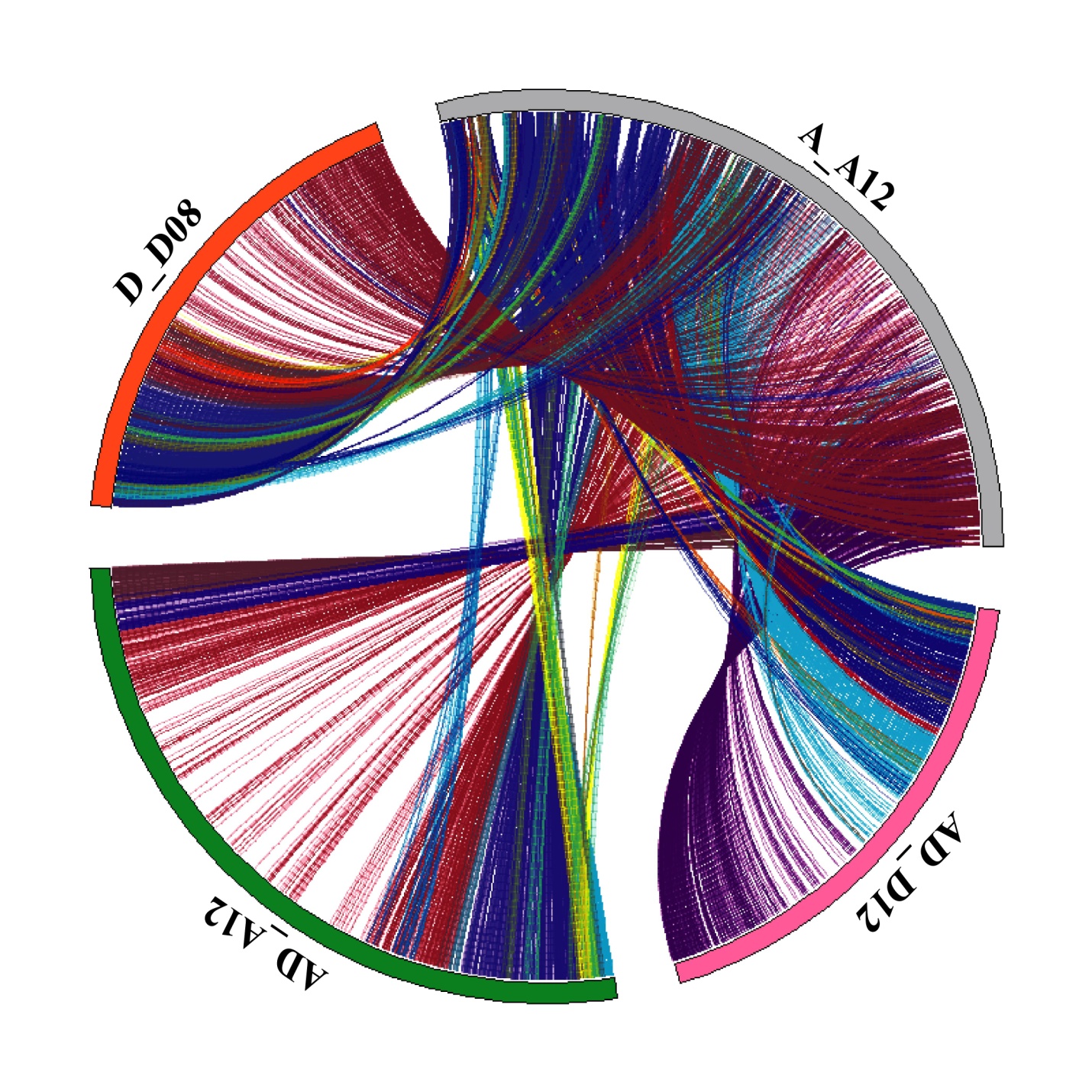


**Fig. S3 Collinearity among homologous chromosomes 12 of three cotton species.**

Collinearity analysis of reassemble *G. arboreum* chromosome 12 (A_A12) with its corresponding homologous chromosomes of *G. raimondii* (D_D08) and *G. hirsutum* (AD_A12 and AD_D12) was performed by MCScan.

**
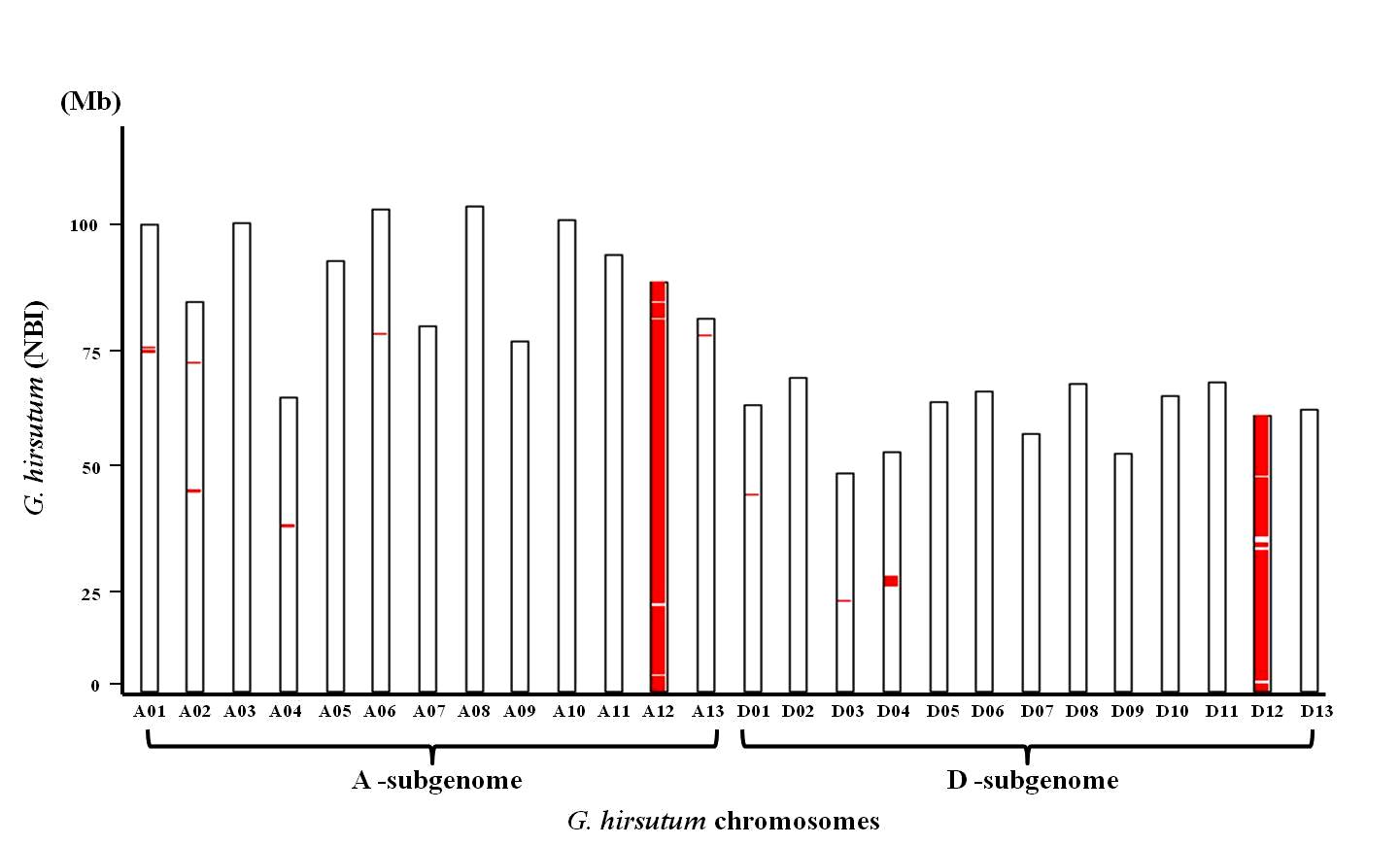
**

**Fig. S4 Alignments of reassembled *G. arboreum* chromosome A_A12 with the whole genome of *G. hirsutum.***

Whole genome of *G. hirsutum* was used as a reference for pair-wise alignment with reassembled chromosome A_A12 of *G. arboreum.* Chromosomes were plotted according to their assembled lengths. White blocks showed the regions which cannot be aligned, while red blocks represented the aligned regions.


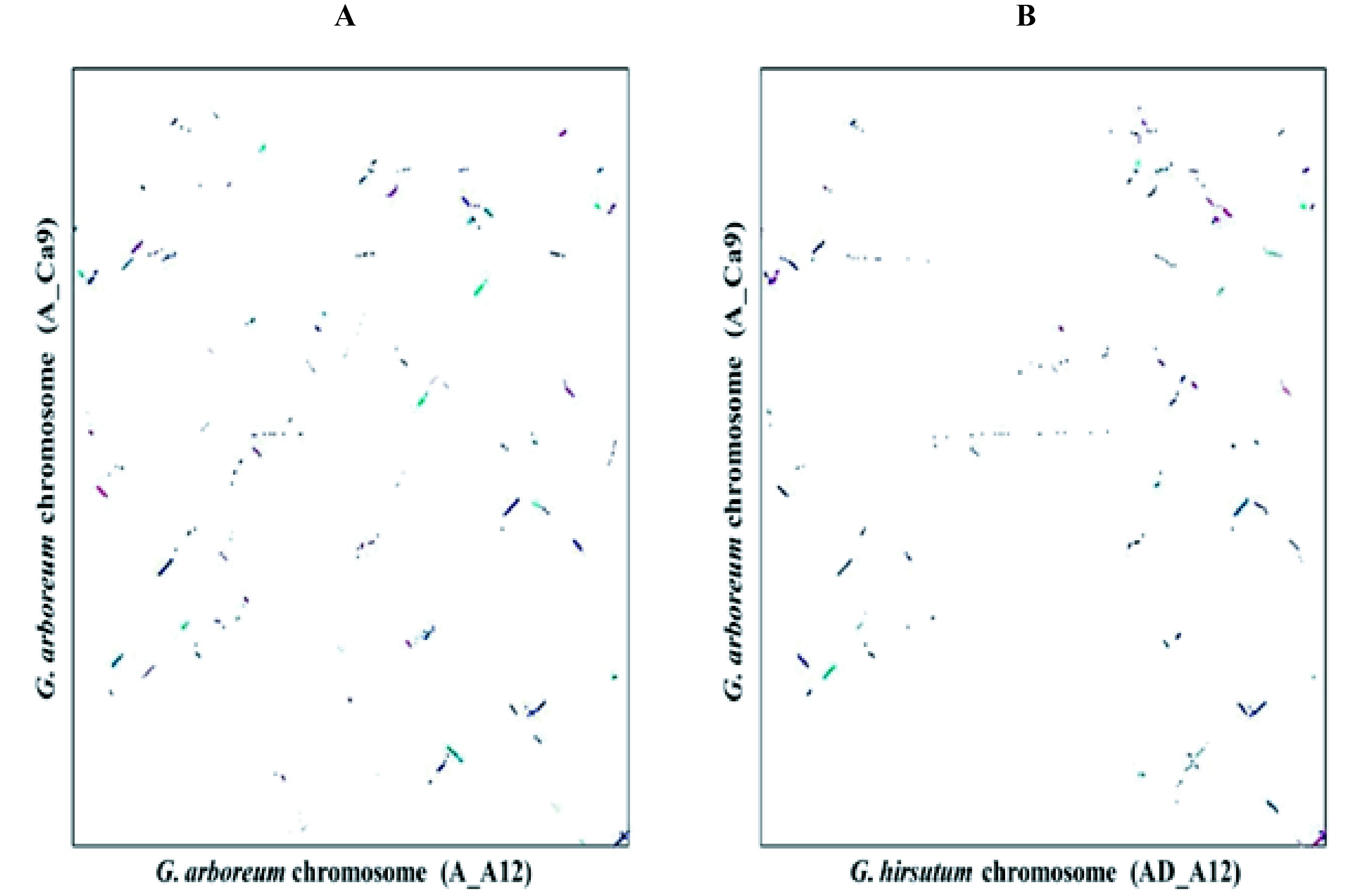


**Fig. S5 Dotplot representation with the previously assembled *G. arboreum* chromosome.**

Previously assembled *G. arboreum* chromosome A_Ca9 (Y-axis) was obtained to depict its collinearity with; **A)** reassembled *G. arboreum* chromosome A_A12 (X-axis), and **B)** *G. hirsutum* chromosome AD_A12 (X-axis). Results showed unobvious collinearity between these chromosomes, which are mainly due to various mis-assemblies in previous assembled *G. arboreum* chromosome A_Ca9.
